# Adverse drug events and contributing factors among pediatric cancer patients at Jimma University medical center, Southwest Ethiopia

**DOI:** 10.1186/s12887-023-03891-9

**Published:** 2023-02-13

**Authors:** Wayessa Olika Tola, Tsegaye Melaku, Diriba Fufa, Tadesse Sheleme

**Affiliations:** 1 Department of Pharmacy, College of Public Health and Medical Science, Mettu University, Mettu, Ethiopia; 2 Department of Clinical Pharmacy, School of Pharmacy, Institute of Health, Jimma University, Jimma, Ethiopia; 3 Department of Pediatrics and Child Health, Institute of Health, Jimma University, Jimma, Ethiopia

**Keywords:** Adverse drug events, Risk factors, Pediatrics, Cancer, Chemotherapy

## Abstract

**Background:**

The characteristics and incidence of adverse drug events (ADEs) among pediatric cancer patients in developing countries have not been well characterized. ADEs & medication errors associated with cancer chemotherapy in children need to be analyzed on their incidence and severity. The purpose of this study was hence, to assess the incidence of adverse drug events and contributing factors among pediatric cancer patients at Jimma university medical center, Jimma, Ethiopia.

**Method:**

A prospective observational method was used to study adverse drug events in pediatrics admitted to the pediatric oncology unit of Jimma University medical center between October and December 2020. The ADEs were identified using multifaceted approaches involving daily chart review, interviews of Parents/caregivers (and/or children themselves), attendance at ward rounds, and voluntary staff reports. Both univariate and multivariate logistic regression were used to assess the predictors of the identified ADEs. Those factors that showed association at *p*-value < 0.25 in the univariate analysis were added to the backward multivariate logistic regression model and the significant association was checked at *p*-value < 0.05.

**Result:**

A total of 73 (46 male and 27 female) patients were included in the study. A total of 466 ADEs were identified with an incidence of 638.36 ADEs per 100 patients, 38.35 ADEs per 100 patient days, and 2.34 ADEs per chemotherapy cycle. The most common ADEs were hematologic toxicities (anemia 55(11.8%), neutropenia 52(11.16%) & thrombocytopenia 31(6.65%)), and gastrointestinal effects (nausea 46(9.87%), vomiting 46(9.87%), anorexia 41(8.8%). Out of 466 ADEs, 150 (32.19%) were classified as common terminology criteria for adverse events (CTCAE) as Grade 1, 199 (42.70%) as Grade 2, 64(13.73%) as Grade 3, 48(10.30%) as grade 4 and 5(1.07%) as Grade 5. Severe acute malnutrition (SAM) is the most common comorbidity present, 20(27.40%) followed by pneumonia, 4(5.50%). Presence of comorbidity (AOR 12.700, CI 1.978–81.549), cancer type (AOR 13.332, CI 3.288–54.059), use of 4 or more chemotherapy drugs (AOR 6.179, CI 1.894–20.165) and length of hospital stay more than 8 days (AOR 5.367, CI 1.167–24.684) were associated with the risk of developing grades 3 and 4 ADEs.

**Conclusion:**

Adverse drug events were common in the pediatric oncology ward of JUMC. In particular, children with multiple chemotherapy drugs and those with the comorbid condition were at greater risk for adverse drug events.

**Supplementary Information:**

The online version contains supplementary material available at 10.1186/s12887-023-03891-9.

## Introduction

An adverse drug event (ADE) refers to any injury caused by a medicine and it encompasses all adverse drug reactions (ADRs), (including allergic or idiosyncratic reactions) as well as medication errors (MEs) that result in harm to a patient [1].

Chemotherapy is considered the primary treatment for childhood cancer [2]. However, they are the most common agents responsible for adverse drug events [3]. This is due in part to the high susceptibility of this group of patients as they have smaller body sizes and larger surface areas than adults [4].

These ADEs pose serious problems to patients in different aspects including hospitalizations so they affect the patient's survival, overall treatment outcomes, morbidity, and mortality rates, and increase substantially the cost of care [5–9].

Several factors have been postulated to contribute to the occurrence of chemotherapy-related adverse events in pediatric cancer patients including the type of chemotherapy involved, the number of chemotherapy, chemotherapy regimens, the dose of chemotherapy, frequency, and duration of administration, and pattern of administration practices is among the factors [10]. This is especially problematic when it comes to low and middle-income counties (LMIC) like Ethiopia because unlike high-income countries (HIC), they lack resources and infrastructure to support intensive treatments leading to increased treatment-related mortality (TRM) and abandonment of care, as well as better supportive care capabilities [11, 12].

Despite the high global burden of chemotherapy-related ADEs concerning mortality, morbidity, patient suffering, health care cost, and overall treatment outcome, there is an insufficient amount of data regarding the magnitude of ADEs occurring in cancer chemotherapy particularly, in pediatric cancer patients in Ethiopia, largely due to lack of an organized and efficient ADR monitoring and reporting program. So the objective of this study is to evaluate the incidence of adverse drug events (ADEs) occurring in pediatric cancer patients treated with chemotherapy at Jimma university medical center.

## Methods

### Study design, setting, and patients

This prospective observational study was conducted at the pediatric oncology unit of Jimma University Medical Center (JUMC), which is found in Jimma town, southwest Ethiopia. The study included all pediatric cancer patients admitted to the pediatric oncology unit of JUMC with a confirmed diagnosis of childhood cancer, who were taking at least one chemotherapy drug. Patients were excluded if they have already completed chemotherapy cycles and if they/their parents were not cooperative to participate.

### Data collection procedure and data quality assurance

Data was collected using a standardized tool adapted from a checklist prepared for the California Health Care Foundation. Different approaches were employed for the data collection which included [13, 14]:

Daily patient chart review for all admissions until discharge/transfer/death: by visiting the study participants daily and reviewing procedure notes, physician progress notes, pertinent laboratory reports, physician orders, medication administration records, nursing/multidisciplinary progress notes, and discharge summaries.

When patients are admitted, all necessary demographic and clinical information of the patients including gender, age of the patient, details of anthropometry, type of cancer diagnosed, number of chemotherapy cycles, types of chemotherapy medications the patient received, history of prior allergy, admission date, the total number of chemotherapy drugs the patient received, chemotherapy regimens, and frequency of administration were recorded using the data collection tool.

Attending ward rounds: the principal investigator attended clinical rounds and asked for the presence of any alerts for ADEs. Interview children and/or parents/caregivers, when further information or clarification of information is required. Pediatric oncology ward staff voluntary reports of any adverse events.

To ensure the quality of the data, the data collectors were trained before the process of data collection. Appropriate supervision and checking were made by the principal investigator to ensure the completeness and consistency of the collected data. All the collected data were checked for completeness and consistency during data management, storage, and analysis.

### Statistical analysis

Data was entered using EpiData version 4.2 and analyzed using Statistical Package for the Social Sciences (SPSS) version 20. Bivariate and multivariable logistic regression analyses were used to assess the significance and strength of the association between the independent variables and dependent variables. A *p*-value of less than 0.05 was considered statistically significant.

### Ethical clearance

Ethical clearance was obtained from the Ethical Review committee of the institute of the health of Jimma University. Written informed assent was obtained from parents/caregivers. For those patients in whom serious ADEs were detected, these were brought to the attention of the responsible staff immediately.

#### Results

### Characteristics of the study population

A total of 84 admitted patients were followed. Among these patients, there 11 patients were excluded (not received any chemotherapy medication). We included 73 patients (46 males & 37 females) of various ages (minimum 6 months, maximum 16 years) for analysis. The mean age of the participants was 7.82(± 4.11 years) and the majority of patients were between 6–11 years of age (27 patients, 37.0%) followed by 3–6 years of age (18 patients, 24.7%). The median weight of the participants was 21.30 (range, 6.6–50.0 kg), and the median height was 121.47 (range, 63-164 cm).

The most common types of cancer diagnosed in patients of both sexes were hematologic malignancies [acute lymphocytic leukemia (ALL) 27(37.0%), Hodgkin’s lymphoma 9(12.3%), anaplastic large cell leukemia (ALCL) 2 (2.7%), Burkett’s lymphoma/diffuse large B-cell lymphoma (DLBCL) 5(6.8%), and acute myeloid leukemia (AML) 5 (6.8%)]. The most common preexisting comorbidity, if any, is severe acute malnutrition (SAM), (27.4%). The majority of parents were farmers. (Table 1).

**Table 1:** Characteristics of pediatric oncology patients and their parents

Variables	Frequency	Percent
**Gender**		
Male	46	63,00
Female	27	37.00
**Age**		
< 3	11	15.07
> 3- < 6	18	24.66
> 6- < 12	27	37.00
> 12	17	23.29
**Weight**		
< 22 kg	42	57.50
> 22 kg	31	42.50
**Height**		
< 119 cm	34	46.60
> 119 cm	39	53.30
**BSA**		
< 0.85m^2^	37	50.70
> 0.85m^2^	36	49.30
**Occupation of parents**		
Farmer	61	83.60
Private business	5	6.80
Employed	4	5.50
House wife	3	4.10
**Types of cancer**		
ALL	27	37.00
HL	9	12.30
AML	5	6.85
DLBCL	5	6.85
ALCL	2	2.74
Rhabdomyosarcoma	5	6.85
Wilm's tumor	8	10.96
Osteosarcoma	5	6.85
Retinoblastoma	2	2.74
Hepatoblastoma	2	2.74
Others	3	4.12
**Comorbidities**		
SAM	20	27.40
Pneumonia	4	5.50
Impaired LFT	1	1.40
N/A	48	65.70

*Abbreviations:* *BSA* body surface area, *LFT* Liver function test, *N/A* Not available, *SAM* severe acute malnutrition, *ALL* acute lymphocytic leukemia, *HL* Hodgkin’s lymphoma, *ALCL* anaplastic large cell leukemia, *AML* acute myeloid leukemia, *DLBCL* diffuse large B-cell leukemia

Others: Nasopharyngeal carcinoma, Ewing sarcoma and Langerhans cell histocytosis

### Chemotherapy regimens administered

A total of 199 cycles of chemotherapies were administered. The majority of patients, (76.70%) had received more than two cycles. 97.26% of patients received two and more two drugs. The chemotherapy regimen comprising vincristine, dexamethasone, 6-mercaptopurine, methotrexate iv/PO, and intrathecal methotrexate (VCR + DEXA + 6-MP + MTX + MTX (IT)) was the most commonly used, 12 (16.44%) followed by a regimen containing, vincristine, actinomycin D and doxorubicin (VAD), 8 (11%). (Table 2).

**Table 2:** Treatment related characteristics of study participants at Jimma university medical center

Variables	Frequency		Percent
**Types of regimens**			
DEXA + VCR + 6-MP + MTX + MTX(IT)	12		16.44
VAD	8		10.96
VCR + DOXO + PDN + L-ASP + MTX(IT)	5		6.85
ABVE-PC	5		6.85
EC	5		6.85
ABVE	4		5.48
VCD	4		5.48
CISP + DOXO	3		4.10
VCR + DOXO + L-ASP + MTX(IT)	2		2.74
VCR + PDN + L-ASP + MTX(IT)	2		2.74
VCR + CARBO + ETOPO	2		2.74
VCR + CYCLO + PDN	2		2.74
Ara-C + TIT	2		2.74
VCR + CYCLO + Ara-C + 6-MP + L-ASP + MTX(IT)	2		2.74
VCR + CYCLO + MTX + TIT	2		2.74
VAC	2		2.74
VCR + 6-MP + MTX(IT)	2		2.74
Other	9		12.33
**Number of chemotherapy drugs**			
1—2 drugs		11	15.00
3 – 5 drugs		52	71.30
≥ 6 drugs		10	13.70
**Number of chemotherapy cycles**			
1 cycle		17	23.30
2 cycles		16	21.90
3 cycles		20	27.40
4 cycles		12	16.40
5 cycles		6	8.30
6 cycles		2	2.70

*Abbreviations:* *VCR* vincristine, *PDN* prednisolone, *ETOPO* etoposide, *TIT* triple intrathecal therapy, *MTX* methotrexate, *IT* intrathecal, *6-MP* 6-mercaptopurine, *L-ASP* L-asparaginase, *Ara-C* arabinosylcytosine (cytarabine), *DOXO* doxorubicin, *CISP* cisplatin, *CARBO* carboplatin, *CYCLO* cyclophosphamide, *ABVE-PC* doxorubicin + bleomycin + vincristine + etoposide + prednisolone + cyclophosamide, *VAD* vincristine + actinomycin D + doxorubicin, *VAC* vincristine + actinomycin D + cyclophosphamide

Other: Ara-C + daunorubicin, vinblastine, etoposide + TIT, carboplatin + doxorubicin

### Characteristics, category, and severity of the ADEs

A total of 466 ADEs were identified in 73 patients. All patients have experienced at least one ADE. The most frequently observed ADEs were hematologic toxicities including [anemia 55(11.80%), neutropenia 52(11.16%), thrombocytopenia 31 (6.65%)] followed by gastrointestinal problems which include [nausea 46 (9.87%), vomiting 46 (9.87%), and anorexia 41(8.8%)], hair loss/alopecia 28(6.0%), and fever and/or chills 39(8.37%).

Among the total ADEs identified, 319 (68.45%) were determined to be category E, 109 (23.39%) were category F, and 33(7.09%) were category H. There were 5(1.07%) deaths determined to be attributed to the ADEs (category I). There was no permanent patient harm reported (category G). According to the WHO-UMC causality assessment, 9.23% of these ADEs were assessed to be certain, 74.67% ADEs were probable/likely and 16.10% were possible. Moreover, based on the national cancer institute common terminology criteria for adverse events version 4.0 (NCI CTCAE) grading system, 32.19% of the ADEs were mild in severity (i.e. grade 1 (*n* = 150)), 42.71% were moderate (i.e. grade 2)), and 24.03% were severe ADEs (grades 3–4 (13.73% grade 3 and 10.3% grade 4)). The rest were grade 5 (Death related to ADE). There were a total of 8 patient deaths during the study, out of which 5(1.07%) were attributed to the ADEs (category I). The most common cause of death during this study was infection (*n* = 5), a complication of the disease (*n* = 2), and an unknown cause (*n* = 1) (Table 3). Severe bacterial/fungal sepsis and severe neutropenic fever were among the infections that led to death.

**Table 3:** Characteristics of the observed ADEs at pediatric oncology unit of Jimma university medical center

Variable	Frequency	Percent
**Types of ADE**		
Anemia	55	11.80
Neutropenia	52	11.16
Thrombocytopenia	31	6.65
Infection	18	3.86
Nausea	46	9.87
Vomiting	46	9.87
Anorexia	41	8.80
Fever	39	8.37
Hair loss	28	6.01
Diarrhea	6	1.29
Constipation	4	0.86
Hepatitis	11	2.36
AST/ALT increased	10	2.15
Creatinine increased	15	3.22
Hyperbilirubinemia	5	1.07
Fatigue	8	1.72
Mucositis	11	2.36
Dry mouth	4	0.86
Skin change	7	1.50
Weight loss	2	0.43
Hematuria	4	0.86
Typhlitis	2	0.43
Other	6	1.29
**Category of ADEs**		
Category E	319	68.45
Category F	109	23.39
Category G	-	-
Category H	33	7.09
Category I	5	1.07
**Severity of ADEs**		
Grade 1	150	32.19
Grade 2	199	42.71
Grade 3	64	13.73
Grade 4	48	10.30
Grade 5	5	1.07
**Probability of ADEs**		
Certain	43	9.23
Probable	75	16.10
Possible	348	74.67

Category E: Error occurred, resulting or contributing to temporary harm to the patient, requiring intervention, Category F: Error occurred, contributing to or resulting in temporary harm to the patient, and requiring initial or prolongation of hospitalization, Category G: Error occurred resulting in permanent patient harm, Category H: Error occurred, requiring intervention to sustain life, and Category I: Error occurred, resulting in death of the patient

Most of the ADEs needed interventions. The most common supportive care given for the developed ADEs is blood transfusion (either whole blood or platelet only), prescription of antiemetics, prescription of additional drugs like antibiotics, holding/discontinuation of chemotherapy drugs, dose/drug modification, increased monitoring of vital signs and/or laboratory values, changing of the IV access site, etc.

### The proportion of severe (grade 3 & 4) ADEs

**Fig. 1:**
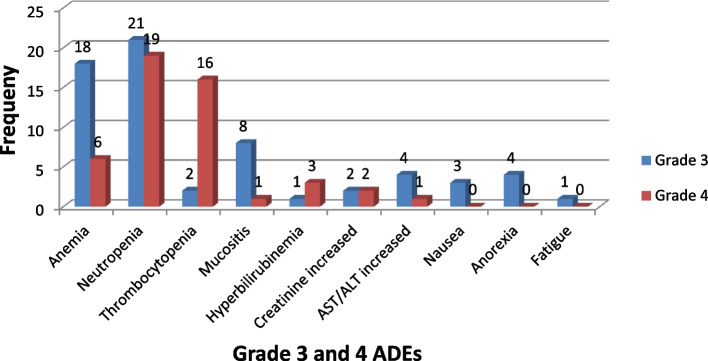
Proportion of grades 3–4 ADEs for the most common ADEs in pediatric cancer patients treated at jimma university medical center, pediatric oncology unit

The most common grade 3–4 events were hematologic events including thrombocytopenia, 30(29.41%), anemia 24(23.53%), and neutropenia 18(17.65%), whereas the most common grades 3–4 nonhematologic events were mucositis 9(8.82%), increased ALT/AST 5(4.90%), increased creatinine 4(3.92%), nausea 4(3.92%), anorexia 4(3.92%) and fatigue 1(0.98%). (Fig. 1).

### ADEs for different cancer types

More than 72% of the total ADEs occurred in patients with blood & lymphoid system cancer (leukemia). Among the blood cancers, higher incidences of ADEs were seen in the patients undergoing treatment for ALL followed by Hodgkin lymphoma (*n* = 156 & 71 respectively). Of the solid tumor cancers, more ADEs were seen in Rhabdomyosarcoma followed by Wilms tumor. (Table 4).

**Table 4:** Adverse drug event occurrence across different cancer types

Diagnosis	Number of ADE	Percent
**Hematology malignancy**		
ALL	156	33.46
HL	71	15.24
AML	43	9.23
DLBCL	36	7.73
ALCL	30	6.44
**Solid tumors**		
Rhabdomyosarcoma	41	8.80
Wilm's tumor	35	7.51
Osteosarcoma	23	4.94
Retinoblastoma	13	2.79
Hepatoblastoma	11	2.36
**Others**	**7**	**1.50**
**Total**	**466**	**100**

*Abbreviations*: *ALL* acute lymphocytic leukemia, *HL* Hodgkin’s lymphoma, *AML* acute myeloid leukemia, *DLBCL* diffuse large B-cell leukemia, *ALCL* anaplastic large-cell leukemia

Others: Nasopharyngeal carcinoma, Ewing sarcoma and Langerhans cell histocytosis

### System organ class (SOC) toxicities concerning chemotherapy cycles

**Fig. 2:**
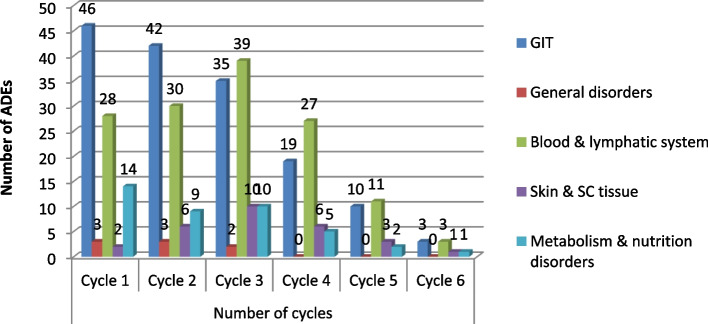
Frequency of different system organ class ADEs according to the number of chemotherapy cycles in which they occurred

Different system organ class (SOC) toxicities occur differently with chemotherapy cycles. Gastrointestinal toxicities like nausea, vomiting, diarrhea, mucositis, and dry mouth consistently decrease with repeated chemotherapy administration as patients adapt to the drugs with repeated exposures. For example, emesis most commonly occurs on the first day of chemotherapy and often persists for several days thereafter. In the same fashion, the frequency of metabolism and nutrition disorders such as anorexia decrease with the number of chemotherapy cycles administered. On the other hand, blood and lymphatic system toxicities do not usually occur immediately after chemotherapy administration, because blood components that have already been produced must be consumed before the effect is evident and the same is true for skin and subcutaneous tissue toxicities like alopecia as hair loss usually begins 7 to 10 days after one treatment, with prominent hair loss noted within 1 or 2 months which usually corresponds to 2nd /3rd cycle of chemotherapy. (Fig. 2).

### ADEs according to various chemotherapy regimens

**Fig. 3:**
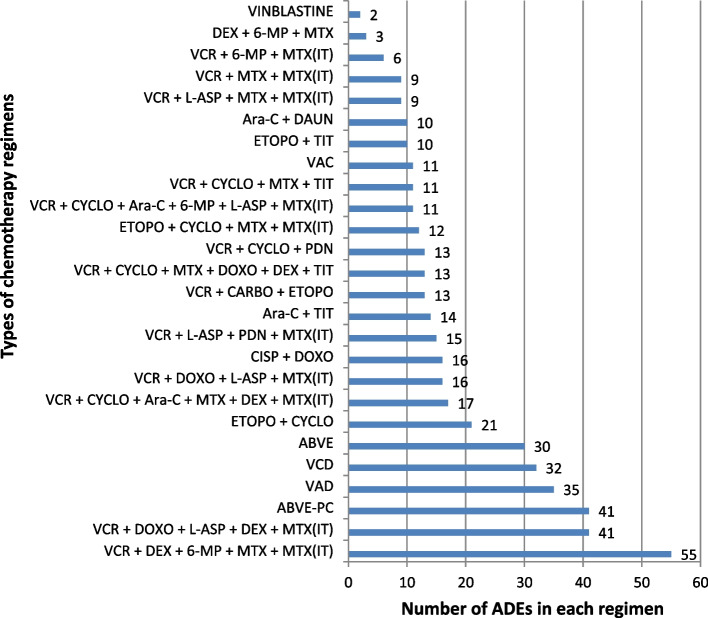
different chemotherapy regimens contributing to ADEs in pediatric cancer patients treated at Jimma university medical center pediatric oncology unit

The most common regimen responsible for the development of adverse drug events was DEXA + VCR + 6-MP + MTX + MTX(IT) followed by a regimen containing VCR + DOXO + PDN + L-ASP + MTX(IT) drugs and ABVE-PC drugs. (Fig. 3).

### *Selected ADEs associated with most suspected regimens*/*containing specific drug*

Some regimens containing specific chemotherapy drugs/classes of chemotherapy are more associated with specific ADEs than other regimens. For example, Regimens containing vincristine are more associated with constipation. Similarly, any chemotherapy regimen containing doxorubicin & cyclophosphamide is associated with alopecia more than any other regimens. (Table 5).

**Table 5:** Selected ADEs associated with specific regimens containing the most suspected agent

Regimens	ADEs				
	**Anemia**	**Neutropenia**	**Thrombo**	**Alopecia**	**Fever**
VCR + DEX + 6-MP + MTX + MTX(IT)	7(12.73)	12(23.08)	3(9.68)	4(14.29)	4(10.0)
VCR + DOXO + L-ASP + DEX + MTX(IT)	5(9.10)	5(9.62)	5(16.13)	2(7.14)	4(10.0)
VCR + DOXO + L-ASP + MTX(IT)	2(3.64)	2(3.85)	2(6.45)	2(7.14)	1(2.50)
VCR + L-ASP + PDN + MTX(IT)	2(3.64)	2(3.85)	2(6.45)	0(0)	2(5.0)
VCR + L-ASP + MTX + MTX(IT)	1(1.82)	1(1.92)	0(0)	1(3.57)	1(2.50)
ETOPO + CYCLO + MTX + MTX(IT)	11.82)	1(1.92)	1(3.22)	1(3.57)	1(2.50)
ABVE	2(3.64)	2(3.85)	1(3.22)	3(10.71)	2(5.0)
ABVE-PC	4(7.27)	4(7.69)	2(6.45)	1(3.57	3(7.50)
ETOPO + CYCLO	2(3.64)	2(3.85)	1(3.22)	2(7.14)	2(5.0)
ETOPO + TIT	1(1.82)	1(1.92)	1(3.22)	0(0)	1(2.50)
DEX + 6-MP + MTX	1(1.82)	1(1.92)	1(3.22)	0(0)	0(0)
VCR + 6-MP + MTX(IT)	1(1.82)	2(3.85)	0(0)	0(0)	1(2.50)
VCR + MTX + MTX(IT)	1(1.82)	1(1.92)	1(3.22)	0(0)	1(2.50)
VAC	0(0)	1(1.92)	0(0)	1(3.57)	1(2.50)
VAD	8(14.55)	2(3.85)	1(3.22)	3(10.71)	5(12.50)
VCD	3(5.45)	2(3.85)	1(3.22)	2(7.14)	2(5.0)
VCR + CYCLO + Ara-C + 6-MP + L-ASP + MTX(IT)	2(3.64)	2(3.85)	1(3.22)	2(7.14)	1(2.50)
VCR + CYCLO + Ara-C + MTX + DEX + MTX(IT)	1(1.82)	1(1.92)	1(3.22)	0(0)	1(2.50)
VCR + CYCLO + MTX + DOXO + DEX + TIT	1(1.82)	1(1.92)	1(3.22)	1(3.57)	1(2.50)
VCR + CYCLO + MTX + TIT	1(1.82)	0(0)	0(0)	0(0)	0(0)
VCR + CARBO + ETOPO	2(3.64)	1(1.92)	0(0)	0(0)	2(5.0)
VCR + CYCLO + PDN	2(3.64)	1(1.92)	2(6.45)	0(0)	1(2.50)
CISP + DOXO	1(1.82)	1(1.92)	1(3.22)	0(0)	0(0)
Ara-C + TIT	2(3.64)	2(3.85)	2(6.45)	2(7.14)	2(5.0)
Ara-C + DAUN	1(1.82)	1(1.92)	1(3.22)	1(3.57)	1(2.50)
VINBLASTINE	1(1.82)	1(1.92)	0(0)	0(0)	0(0)
TOTAL	55(100)	52(100)	31(100)	28(100)	40(100)

*Abbreviations:* *VAC* vincristine + actinomycin D + cyclophosphamide, *VCD* vincristine + cyclophosphamide + doxorubicin, *VAD* vincristine + actinomycin D + doxorubicin; *ABVE/ABVE-PC* adriamycin + bleomycin + vincristine + etoposide + prednisolone + cyclophosphamide

### Incidence of ADEs

Seventy-three patients received a total of 199 chemotherapy cycles and developed a total of 466 ADEs with a mean of 6.38 (± 3.65). All patients on chemotherapy have experienced at least one ADE. The number of ADEs per individual patient ranged from a minimum of 1 ADE to a maximum of 17 ADEs. A total of 1215 patient days were recorded with the mean length of hospital stay 16.64(± 13.3) with a range of 3–90 days. The cumulative incidence of ADEs was calculated per 100 admissions, per 100 patient days and a per cycle of chemotherapy administered. Accordingly, the estimated incidence of these 466 ADEs based on 73 patient records was 638.36 ADEs per 100 patients, 38.35 ADEs per 100 patient days, and 2.34 ADEs per chemotherapy cycle.

### Incidence of ME

A total of 51 medication errors have been identified in 73 patients with 199 total numbers of admissions and 1215 total patient days giving the cumulative incidence of 69.86 MEs per 100 patients, 25.63 MEs per 100 admissions, 41.98 MEs per 1000 patient days, and 0.26 ME per chemotherapy cycle.

### Factors associated with the grade 3 and 4 ADEs

In the results of the full multivariate model analysis, the presence of comorbidity (AOR 12.700, CI 1.978–81.549), cancer type (AOR 13.332, CI 3.288–54.059), use of 4 or more chemotherapy drugs (AOR 6.179, CI 1.894–20.165) and length of hospital stay more than 8 days (AOR 5.367, CI 1.167–24.684). were associated with the risk of developing grades 3 and 4 ADEs. (Table 6).

**Table 6:** Multivariable logistic regression results for factors associated with the occurrence of ADEs pediatrics with cancer at JUMC

Variables	Category	Grade 3—4 ADEs		COR(95% CI)	*P* value	AOR (95% CI)	*P* value
		No	Yes				
**Sex**	Female	14	13	1.0			
	Male	14	32	2.462(0.922–6.572)^*^	0.072	3.233(0.816–12.806)	0.095
**Age(years)**	≥ 12	6	11	1.0			
	6–11	10	17	3.208(0.660–15.587)	0.148		
	3–6	5	13	2.975(0.694–12.756)	0.142		
	< 3	7	4	4.550(0.915–22.627)	0.064		
**Presence of comorbidity**	No	26	22	1.0			
	Yes	2	23	13.591(2.878–64.190)^*^	0.001	12.700(1.978–81.549)^**^	0.007
**Risk group**	Standard risk	7	3	1.0			
	High risk	9	19	1.091(0.182–6.555)	0.924		
**Cancer type**	Non leukemic	18	7	1.0			
	Leukemic	10	38	9.771(3.198–29.855)^*^	0.000	13.332(3.288–54.059)^**^	0.000
**Number of CT cycle**	1—2 cycle	19	14	1.0			
	≥ 3 cycle	18	22	0.676(0.260–1.762)	0.424		
**Number of CT Drugs**	1 – 3	19	13	1.0		1.0	
	≥ 4	9	32	5.197(1.870–14.440)^*^	0.002	6.179(1.894–20.165)^**^	0.003
**Length of hospital stay**	1 – 7	12	6	1.0		1.0	
	≥ 8	16	39	4.875(1.560–15.239)^*^	0.006	5.367(1.167–24.684)^**^	0.031

*Abbreviations:* *COR* Crude odds ratio, *AOR* Adjusted Odds Ratio, *CT* Chemotherapy

^*^ Significant association; *p* < 0.25

^**^ Significant association; *p* < 0.05

## Discussion

This prospective observational study has attempted to assess the incidence of adverse drug events and associated factors among pediatric cancer patients treated at Jimma university medical center, pediatric oncology unit, Jimma, Ethiopia.

In this study, all patients receiving cancer chemotherapy experienced at least one ADE with an estimated incidence of 6.38 ADEs per patient, and 38.35 per 100 patient days. When compared with prior studies, it is higher than those incidences reported in other studies. Takata et al. [15] reported 15.7 ADEs per 1000 patient days, Kaushal et al. [16] reported 6.6 ADEs per 1000 patient days, and Sakuma et al. [17] reported 37.8 ADEs per 1000 patient-days, but lower than that reported by Koizumi et al. [18] (7.1 ADEs incidence per cancer patient). This variation might be due to differences in the trigger to which the event was searched, the methodology & definition employed, the background of the study population, the study setting, and local practices/trends.

Regarding the severity of ADEs in this study, 98.93% caused temporary harm to the patient. A study that used a similar method reported that all of the ADEs they detected caused temporary harm [15]. On the other hand, 1.07% of the ADEs in our study resulted in death. This finding is comparatively lower than a study done in the general pediatric ward of the same hospital, where 9% of ADEs resulted in permanent harm/death [19]. This could be due to difference in the background of the patients, sample size and more potential medication errors that lead to ADEs in the general pediatric ward. Moreover, according to CTCAE, 74.89% of the ADEs were grade 1 and 2, while 25.11% of them were grade 3 to 5, which is comparable to a study by Parande et al. [4] However, it is not in line with a prior study conducted at Gondar University Referral Hospital Oncology Centre, where 70.1% of the reported ADEs were grade 3–5 and the rest 29.9% were grade 1 and 2 [20].

In our study, hematologic toxicities like anemia, neutropenia & thrombocytopenia were the most common and dose-limiting ADEs, leading to potentially life-threatening complications. Moreover, anemia was the most commonly encountered hematologic ADE, (39.57%) which is nearly similar to the finding of two other studies. [21, 22] It was seen in this study that the drug regimen comprising DEX + VCR + 6-MP + MTX + MTX (IT), used for the treatment of acute lymphoblastic leukemia, was the most contributed to hematological toxicities.

Our study identified risk factors associated with the development of severe ADEs. Particularly, SAM was found a distinctive problem in our setting and is associated with grade 3–4 ADEs. A similar study also reported that SAM increases treatment-related toxicity in cancer patients [23, 24]. This could be due to lower protein binding as a result of a lower level of serum albumin in malnourished patients leading to a high level of free drugs. Therefore, every effort/nutrition support should be used to manage/improve the nutritional status of malnourished patients before the use of chemotherapy in such patients.

The number of chemotherapy drugs used was found to be associated with the occurrence of ADEs in this study. A systematic review of 26 studies involving 85,212 patients confirmed that the number of drugs was an independent risk factor for ADEs. [25] This might be due to the extra risk of ADEs when receiving more chemotherapy drugs. More severe ADEs were seen in patients undergoing treatment for blood and lymphoid malignancy (leukemic cancer). This agrees with other studies [26, 27]. This might be due to the use of more complex regimens of chemotherapy for relatively long duration and intensive treatment required in such patients than in non-leukemic cancer patients.

This study has also limitations. It is a single-center study with a small sample and therefore might not be generalized to other centers in Ethiopia. The incidence of ADEs might have been underestimated as some laboratory services were not available and thus ADEs may have not been detected. Any event that might have occurred in patients between the treatment cycles was not monitored as the patients went home after receiving one chemotherapy cycle until the next chemotherapy cycle but it was unlikely that we missed those events as such events would be reported by the patients upon returning to the hospital.

## Conclusion

The results of this study suggests that ADEs are common in the pediatric oncology unit of jimma university medical center. Hematologic toxicities resulting from cancer chemotherapy in pediatrics are found to be the most severe and potentially life threatening ADEs and DEX + VCR + 6-MP + MTX + MTX (IT) was the most common regimen contributed to hematological toxicities. Severe ADEs were found to occur more likely in pediatrics with leukemic cancer, more number of chemotherapy medication, the presence of comorbidity. Eventhough majority of the ADEs caused temporary harm to the patients, there were patient deaths as a result of some ADEs which needs to search strategies for future prevention/minimize this consequences.

## Supplementary Information

**Additional file 1.** 

**Additional file 2.** 

## Data Availability

The data that were used and/or analysed during the current study are not publicly available due to patient privacy, but are available from the corresponding author on reasonable request.

## Abbreviations

ADE: Adverse Drug Event
AOR: Adjusted odds ratio
CI: Confidence Interval
NCC MERP: National Coordinating Council for Medication Error Reporting and Prevention
NCI CTCAE: National cancer institute common terminology criteria for adverse events
WHO-UMC: World Health Organization- Uppsala Monitoring Centre
