# Supplementary material for: Adverse drug events and contributing factors among pediatric cancer patients at Jimma University medical center, Southwest Ethiopia
Source: BMC Pediatr. 2023 Feb 13;23:77. doi: 10.1186/s12887-023-03891-9 (PMC9923905; doi:10.1186/s12887-023-03891-9)
Supplement: Supplementary file 1 — Additional file 1. [file 12887_2023_3891_MOESM1_ESM.docx]

**Summary of supportive** **managements for pediatric cancer patients at JUMC, pediatric oncology unit**

In addition to chemotherapy drugs, the patients were also prescribed some premedications to prevent the expected ADRs of chemotherapeutic agents. The most commonly prescribed premedications include: Ondansetron, dexamethasone, metoclopramide and Mesna either alone or in combination. Anti-invectives were also prescribed, mainly for the management of some of the observed ADEs, including vancomycin, Ceftazidime, cotrimoxazole, ciprofloxacin, ceftriaxone and gentamycin. Antifungals, such as fluconazole and Miconazole oral gel were also used as a management or prophylaxis against most causative organisms.

| **Management** | **Reason** | **Drugs used** |
| --- | --- | --- |
| **Hydration*** | To facilitate drug excretion | IV fluids at 3000ml/m2/day (normal saline), before & after chemotherapy administration |
| **Antiemetics**** | To prevent/reduce chemotherapy induced nausea and vomiting(CINV) | 1. **Ondansetron**  5mg/m^2^ IV/po prechemotherapy and then q8h until 24 hours after last dose of chemotherapy or until no longer nauseated  **2. Metoclopramide** 1mg/kg /dose po/IV q8h until 24 hours after last dose chemo  3. **Dexamethasone** 6mg/m^2^/dose po/IV q8H until 24 hrs after last dose chemo |
| **Prophylactic medication** | To reduce risk of metabolic problems associated with tumour lysis | Allopurinol 400mg/m2 /day in 3 divided doses orally for 5 days starting 24 hours before first prednisone dose |
|  | PCP prophylaxis | **Cotrimoxazole** 5 - 10mg/kg/day PO divided in two doses, given on 3 consecutive days every week |
|  | To prevent hemorrhagic cystitis (usually associated with cyclophosphamide & ifosphamide) | Mesna (200 mg/m^2^/dose) IV |
|  | To prevent chemical conjunctivitis (usually associated with cytarabine) | dexamethasone ophthalmic drops |
|  | To prevent/treat fungal infection | Fluconazole 5 - 10 mg/kg/day, PO, once a day |
|  | To reduce/treat risk for infections | - Ciprofloxacillin 10mg/kg/dose (250 a 350 mg/m2/dose) IV or PO every 12 hours - Vancomycin: 10mg/kg/dose (400 mg/m2/dose) IV every 12 hours |
| **Nutritional supplements** | For malnutrition | RUTF  F-100  F-75 |

* Timing of administration varies with different chemotherapy agents

**based on the emetogenicity of chemotherapy drugs used
