# Supplementary material for: Adverse drug events and contributing factors among pediatric cancer patients at Jimma University medical center, Southwest Ethiopia
Source: BMC Pediatr. 2023 Feb 13;23:77. doi: 10.1186/s12887-023-03891-9 (PMC9923905; doi:10.1186/s12887-023-03891-9)
Supplement: Supplementary file 2 — Additional file 2. [file 12887_2023_3891_MOESM2_ESM.docx]

**ANNEX I. DATA COLLECTION TOOL**

1. Name…………………… Age ……… Sex……. Card No……………………
2. Weight…………height …….. BSA……. co-morbidity…………………………..
3. Occupation of the parents:
4. Farmer
5. Private Business
6. Employed
7. House wife
8. Diagnosis ……………………………………………….. Stage …………… GFR…….
9. **Laboratory tests done**

| Variable | Cycle 1 | Cycle 2 | Cycle 3 | Cycle 4 | Cycle 5 | Cycle 6 |
| --- | --- | --- | --- | --- | --- | --- |
| Weight(kg) |  |  |  |  |  |  |
| BP |  |  |  |  |  |  |
| Hgb |  |  |  |  |  |  |
| WBC |  |  |  |  |  |  |
| Neutrophil |  |  |  |  |  |  |
| Platelet |  |  |  |  |  |  |
| ALT/AST |  |  |  |  |  |  |
| Tot bil/direct bilirubin |  |  |  |  |  |  |
| Creatinine |  |  |  |  |  |  |

1. **Treatments given**

| Medication | | Dose | | Administration | | |
| --- | --- | --- | --- | --- | --- | --- |
| Pre chemo IV fluid |  |  | |  | | |
| Antiemetic |  |  | |  | | |
|  |  |  | |  | | |
|  |  |  | |  | | |
| Prophylactic drug |  |  | |  | | |
| Chemotherapy |  | | | | | |
|  | Cycle 1 | Cycle 2 | Cycle 3 | Cycle 4 | Cycle 5 | Cycle 6 |
|  |  |  |  |  |  |  |
|  |  |  |  |  |  |  |
|  |  |  |  |  |  |  |
|  |  |  |  |  |  |  |
|  |  |  |  |  |  |  |
|  |  |  |  |  |  |  |
| Post CT fluid |  | Dose | | Administration | | |
|  |  |  | |  | | |
| Antiemetic |  |  | |  | | |
|  |  |  | |  | | |
|  |  |  | |  | | |

1. Other diseases (comorbidities) present:
2. SAM_____
3. Pneumonia______
4. Impaired RFT_____
5. Impaired LFT_____
6. Other, specify_____
7. Complication/ADR from chemotherapy

| **ADE** | Cycle 1 | Cycle 2 | Cycle 3 | Cycle 4 | Cycle 5 | Cycle 6 |
| --- | --- | --- | --- | --- | --- | --- |
| Anorexia |  |  |  |  |  |  |
| Constipation |  |  |  |  |  |  |
| Dehydration |  |  |  |  |  |  |
| Diarrhea |  |  |  |  |  |  |
| Fatigue |  |  |  |  |  |  |
| Fever |  |  |  |  |  |  |
| Hair Loss |  |  |  |  |  |  |
| Thrombocytopenia |  |  |  |  |  |  |
| Anemia |  |  |  |  |  |  |
| Neutropenia |  |  |  |  |  |  |
| Mucositis |  |  |  |  |  |  |
| Dry Mouth |  |  |  |  |  |  |
| Nausea |  |  |  |  |  |  |
| Vomiting |  |  |  |  |  |  |
| Peripheral neurotoxicity |  |  |  |  |  |  |
| Skin Changes |  |  |  |  |  |  |
| Weight Loss |  |  |  |  |  |  |
| Weight gain |  |  |  |  |  |  |
| Hemorrhagic cystitis |  |  |  |  |  |  |
| Anaphylaxis |  |  |  |  |  |  |
| Pancreatitis |  |  |  |  |  |  |
| Hepatitis |  |  |  |  |  |  |
| Other |  |  |  |  |  |  |

**Pediatric ADE Patient Record Review Sheet**

ADE found: Yes_______ NO___________

Describe the adverse drug event: _____________________________________________________________________________________________

Date the event started: __________

Date the event stopped: ___________

Any relevant history, Allergies, Previous exposure, Baseline test results/lab data, hepatic/renal dysfunction,etc.____________________________________________________________________________________________________________

Medication involved or suspected to cause ADE: (Name, dose, route, frequency, indication, date started) _____________________________________

**ADE outcome:**

1. Intervention required to prevent permanent impairment or damage_________
2. Allergy______
3. Disability ______
4. Life threatening situation_______
5. Hospitalization prolonged _______
6. Death:________
7. Other outcome (describe) __________________________________________

**Management of ADE: YES__________ NO______________**

If yes, tick in the space for interventions taken in response to ADE:

1. Administration of antidote/reversal agent ________
2. Medication dose changed ________
3. Medication D/C ____________
4. Required increased monitoring (Lab or V/S) _________________
5. Transfer to higher level care______________
6. Other intervention _________________________________________

**Harm Category of the ADE (adapted from NCC MERP):**

Category E: Temporary harm to the patient and required intervention

Category F: Temporary harm to the patient and required initial or prolonged hospitalization

Category G: Permanent patient harm

Category H: Intervention required to sustain life

Category I: Patient death

**Annex II: Grading of Common-Toxicity-Criteria according to NCI, modified by SIOP**

| Adverse Event | Grade | | | | |
| --- | --- | --- | --- | --- | --- |
|  | 1 | 2 | 3 | 4 | 5 |
| Anemia | Hemoglobin (Hgb) <LLN - 10.0 g/dL; <LLN - 6.2 mmol/L;  <LLN - 100 g/L | Hgb <10.0 - 8.0 g/dL; <6.2 - 4.9 mmol/L; <100 - 80g/L | Hgb <8.0 g/dL; <4.9 mmol/L;  <80 g/L; transfusion indicated | Life-threatening  consequences; urgent intervention indicated | Death |
| Febrile neutropenia | - | - | ANC <1000/mm3 with a single temperature of >38.3 degrees C (101 degrees F) or a  sustained temperature of  >=38 degrees C (100.4  degrees F) for more than one  hour. | Life-threatening  consequences; urgent  intervention indicated | Death |
| Constipation | Occasional or intermittent symptoms; occasional use of stool softeners, laxatives, dietary modification, or enema | Persistent symptoms with regular use of laxatives or enemas; limiting instrumental ADL | Obstipation with manual evacuation indicated; limiting self care ADL | Life-threatening consequences; urgent intervention indicated | Death |
| Diarrhea | Increase of <4 stools per day over baseline; mild increase in ostomy output compared to baseline | Increase of 4 - 6 stools per day over baseline; moderate increase in ostomy output compared to baseline | Increase of >=7 stools per day over baseline; incontinence; hospitalization indicated; severe increase in ostomy output compared to baseline; limiting self care ADL | Life-threatening consequences; urgent intervention indicated | Death |
| Dry mouth | Asymptomatic; clinical or  diagnostic observations only; intervention not indicated | Symptomatic; altered GI function | Severely altered GI function; tube feeding, TPN or hospitalization indicated; elective operative intervention indicated | Life-threatening consequences; urgent intervention indicated | Death |
| Mucositis oral | Asymptomatic or mild symptoms; intervention not indicated | Moderate pain; not interfering with oral intake; modified diet indicated | Severe pain; interfering with oral intake | Life-threatening consequences; urgent intervention indicated | Death |
| Nausea | Loss of appetite without alteration in eating habits | Oral intake decreased without significant weight loss, dehydration or malnutrition | Inadequate oral caloric or fluid intake; tube feeding, TPN, or hospitalization indicated | - | - |
| Vomiting | 1 - 2 episodes (separated by 5 minutes) in 24 hrs | 3 - 5 episodes (separated by 5 minutes) in 24 hrs | >=6 episodes (separated by 5 minutes) in 24 hrs; tube feeding, TPN or hospitalization indicated | Life-threatening consequences; urgent intervention indicated | Death |
| Fatigue | Fatigue relieved by rest | Fatigue not relieved by rest; limiting instrumental ADL | Fatigue not relieved by rest, limiting self care ADL | - | - |
| Fever | 38.0 - 39.0 degrees C (100.4 - 102.2 degrees F) | >39.0 - 40.0 degrees C (102.3 - 104.0 degrees F) | >40.0 degrees C (>104.0 degrees F) for <=24 hrs | >40.0 degrees C (>104.0 degrees F) for >24 hrs | Death |
| Infusion related reaction | Mild transient reaction; infusion interruption not indicated; intervention not indicated | Therapy or infusion interruption indicated but responds promptly to symptomatic treatment (e.g., antihistamines, NSAIDS, narcotics, IV fluids); prophylactic medications indicated for <=24 hrs | Prolonged (e.g., not rapidly responsive to symptomatic medication and/or brief interruption of infusion); recurrence of symptoms following initial improvement; hospitalization indicated for clinical sequelae | Life-threatening consequences; urgent intervention indicated | Death |
| Infusion site extravasation | - | Erythema with associated symptoms (e.g., edema, pain, induration, phlebitis) | Ulceration or necrosis; severe tissue damage; operative intervention indicated | Life-threatening consequences; urgent intervention indicated | Death |
| Injection site reaction | Tenderness with or without associated symptoms (e.g., warmth, erythema, itching) | Pain; lipodystrophy; edema; phlebitis | Ulceration or necrosis; severe tissue damage; operative intervention indicated | Life-threatening consequences; urgent intervention indicated | Death |
| Pain | Mild pain | Moderate pain; limiting instrumental ADL | Severe pain; limiting self care ADL | - | - |
| Allergic reaction | Transient flushing or rash, drug fever <38 degrees C (<100.4 degrees F); intervention not indicated | Intervention or infusion interruption indicated; responds promptly to symptomatic treatment (e.g., antihistamines, NSAIDS, narcotics); prophylactic medications indicated for <=24 hrs | Prolonged (e.g., not rapidly responsive to symptomatic medication and/or brief interruption of infusion); recurrence of symptoms following initial improvement; hospitalization indicated for clinical sequelae (e.g., renal impairment, pulmonary infiltrates) | Life-threatening consequences; urgent intervention indicated | Death |
| Anaphylaxis | - | - | Symptomatic bronchospasm, with or without urticaria; parenteral intervention indicated; allergy-elated edema/angioedema; hypotension | Life-threatening consequences; urgent intervention indicated | Death |
| Neutrophil count decreased | <LLN - 1500/mm3; <LLN - 1.5 x 10e9 /L | <1500 - 1000/mm3; <1.5 - 1.0 x 10e9 /L | <1000 - 500/mm3;  <1.0 - 0.5 x 10e9 /L | <500/mm3;  <0.5 x 10e9 /L | - |
| Platelet count decreased | <LLN - 75,000/mm3; <LLN - 75.0 x 10e9 /L | <75,000 - 50,000/mm3; <75.0 - 50.0 x 10e9 /L | <50,000 - 25,000/mm3; <50.0 - 25.0 x 10e9 /L | <25,000/mm3; <25.0 x 10e9/L | - |
| Weight gain | 5 - <10% from baseline | 10 - <20% from baseline | >=20% from baseline | - | - |
| Weight loss | 5 to <10% from baseline; intervention not indicated | 10 - <20% from baseline; nutritional support indicated | >=20% from baseline; tube feeding or TPN indicated | - | - |
| White blood cell decreased | <LLN - 3000/mm3; <LLN - 3.0 x 10e9 /L | <3000 - 2000/mm3; <3.0 - 2.0 x 10e9 /L | <2000 - 1000/mm3; <2.0 - 1.0 x 10e9 /L | <1000/mm3; <1.0 x 10e9 /L | - |
| Dehydration | Increased oral fluids indicated; dry mucous membranes; diminished skin turgor | IV fluids indicated <24 hrs | IV fluids or hospitalization indicated | Life-threatening consequences; urgent intervention indicated | Death |
| Anorexia | Loss of appetite without alteration in eating habits | Oral intake altered without significant weight loss or malnutrition; oral nutritional supplements indicated | Associated with significant weight loss or malnutrition (e.g., inadequate oral caloric and/or fluid intake); tube feeding or TPN indicated | Life-threatening consequences; urgent intervention indicated | Death |
| Alopecia | Hair loss of <50% of normal for that individual that is not obvious from a distance but only on close inspection; a different hair style may be required to cover the hair loss but it does not require a wig or hair piece to camouflage | Hair loss of >=50% normal for that individual that is readily apparent to others; a wig or hair piece is necessary if the patient desires to completely camouflage the hair loss; associated with psychosocial impact | - | - | - |
| Skin hyperpigmentation | Hyperpigmentation covering <10% BSA; no psychosocial impact | Hyperpigmentation covering >10% BSA; associated psychosocial impact | - | - | - |
| Skin hypopigmentation | Hypopigmentation or depigmentation covering <10% BSA; no psychosocial impact | Hypopigmentation or depigmentation covering >10% BSA; associated psychosocial impact | - | - | - |
| Stevens-Johnson syndrome | - | - | Skin sloughing covering <10% BSA with associated signs (e.g., erythema, purpura, epidermal detachment and mucous membrane detachment) | Skin sloughing covering 10 - 30% BSA with associated signs (e.g., erythema, purpura, pidermal detachment and mucous membrane detachment) | Death |
| Hypertension | Prehypertension (systolic BP 120 - 139 mm Hg or diastolic BP 80 - 89 mm Hg) | Stage 1 hypertension (systolic BP 140 - 159 mm Hg or diastolic BP 90 - 99 mm Hg); medical intervention indicated; recurrent or persistent (>=24 hrs); symptomatic increase by >20 mm Hg (diastolic) or to >140/90 mm Hg if previously WNL; monotherapy indicated Pediatric: recurrent or persistent (>=24 hrs) BP >ULN; monotherapy indicated | Stage 2 hypertension (systolic BP >=160 mm Hg or diastolic BP >=100 mm Hg); medical intervention indicated; more than one drug or more intensive therapy than previously used indicated Pediatric: Same as adult | Life-threatening consequences (e.g., malignant hypertension, transient or permanent neurologic deficit, hypertensive crisis); urgent intervention indicated Pediatric: Same as adult | Death |
| Hypotension | Asymptomatic, intervention not indicated | Non-urgent medical intervention indicated | Medical intervention or hospitalization indicated | Life-threatening and urgent intervention indicated | Death |
